# Real-World Treatment Outcomes Associated With Early Versus Delayed Vedolizumab Initiation in Patients With Ulcerative Colitis

**DOI:** 10.1093/crocol/otae061

**Published:** 2024-10-22

**Authors:** Noa Krugliak Cleveland, Ninfa Candela, John A Carter, Maja Kuharic, Joyce Qian, Zhaoli Tang, Robin Turpin, David T Rubin

**Affiliations:** University of Chicago Medicine, Inflammatory Bowel Disease Center, Chicago, IL, USA; Takeda Pharmaceuticals USA, Inc., Lexington, MA, USA; OPEN Health, Bethesda, MD, USA; Department of Pharmacy Systems, Outcomes and Policy, University of Illinois Chicago, Chicago, IL, USA; OPEN Health, Bethesda, MD, USA; OPEN Health, Bethesda, MD, USA; Takeda Pharmaceuticals USA, Inc., Lexington, MA, USA; University of Chicago Medicine, Inflammatory Bowel Disease Center, Chicago, IL, USA

**Keywords:** inflammatory bowel disease, ulcerative colitis, vedolizumab, treatment sequence, early treatment

## Abstract

**Background:**

Patients with ulcerative colitis (UC) typically receive a targeted inflammatory bowel disease therapy after treatment with conventional therapies and after the development of significant morbidity. Evidence suggests that early biologic treatment after diagnosis could improve treatment response and prevent disease complications compared with delayed biologic treatment after conventional therapy.

**Methods:**

RALEE was a retrospective study using claims data from IBM® MarketScan® Research Databases between January 1, 2016 and December 31, 2019. Adults with UC and at least one claim for vedolizumab were categorized into Early or Delayed Vedolizumab groups according to whether they had received vedolizumab within 30 days of diagnosis or after conventional therapy (5-aminosalicylates, corticosteroids, and immunomodulators), respectively. Treatment response was assessed at 2, 6, and 12 months after vedolizumab treatment initiation and was analyzed with logistic regression (bivariate).

**Results:**

At 2 months, Delayed Vedolizumab was associated with significantly higher odds of nonresponse than Early Vedolizumab (odds ratio [OR], 2.509; 95% confidence interval [CI], 1.28-4.90). Delayed Vedolizumab was not significantly associated with odds of nonresponse at 6 months (OR, 1.173; 95% CI, 0.72-1.90) or at 12 months (OR, 0.872; 95% CI, 0.55-1.37). Mean total healthcare costs were similar in the Early Vedolizumab ($6492) and Delayed Vedolizumab ($5897) groups, although there were small differences in costs from different types of claims.

**Conclusions:**

Patients who received vedolizumab early after UC diagnosis were less likely to experience nonresponse at 2 months and incurred similar healthcare costs at 12 months compared with patients who received delayed vedolizumab.

## Graphical Abstract

**Graphical Abstract:**
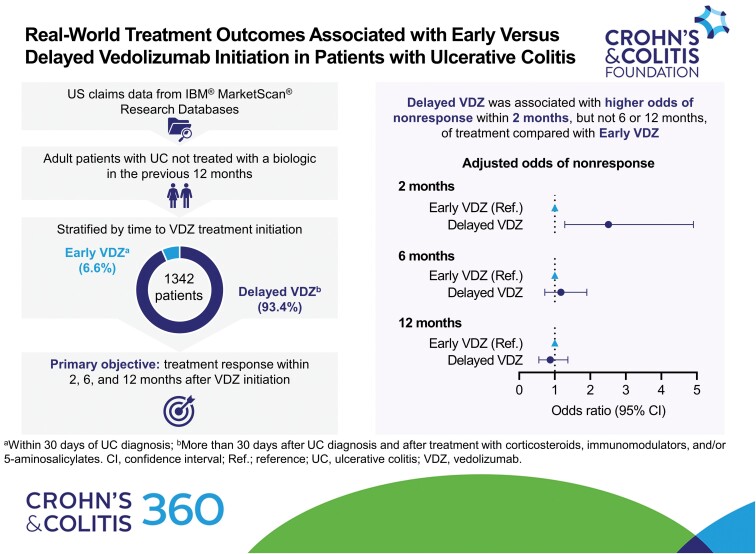

**Key Messages**

**What Is Known**

- Early treatment with a biologic may improve treatment outcomes in patients with Crohn’s disease; evidence is limited in patients with ulcerative colitis.

**What Is New Here**

- In patients with ulcerative colitis, delayed initiation of vedolizumab was associated with a higher likelihood of nonresponse within 2 months after vedolizumab treatment initiation than early vedolizumab.
- Healthcare costs at 12 months were similar between patients who received early or delayed vedolizumab.

**How Can This Study Help Patient Care**

- The results of these analyses suggest that patients with ulcerative colitis who receive vedolizumab early after their diagnosis may have better response within 2 months than those who initiated vedolizumab after treatment with conventional therapy, supporting its use of vedolizumab as an induction therapy.

## Introduction

Ulcerative colitis (UC) is a chronic and progressive inflammatory bowel disease (IBD), characterized by inflammation of the colon and rectum, with a relapsing and remitting course.^1,2^ Symptoms of UC include abdominal pain, diarrhea, fecal urgency, and even bowel incontinence.^3^ If uncontrolled, UC can lead to compromised bowel structure and function, surgery, and increased risk of cancer.^1,4–8^

Conventional therapies for UC include 5-aminosalicylates (5-ASA), corticosteroids, and immunomodulators; however, these therapies have some limitations. The efficacy of 5-ASA is limited to mild disease; corticosteroids are associated with numerous adverse events; and immunomodulators increase the risk of serious infection or lymphoma.^9–14^ Furthermore, conventional therapies target UC symptoms and are not disease-modifying.^15^

Several targeted IBD therapies, including small molecules and biologics, such as the α_4_β_7_ integrin inhibitor vedolizumab, are approved for the treatment of moderately to severely active UC.^16^ The aims of treatment with a targeted IBD therapy are to improve clinical symptoms, attain mucosal healing, and, ultimately, prevent disease progression; however, in clinical practice, targeted IBD therapies are often used after conventional therapies have failed, even in patients who initially present with moderately to severely active UC. This delay in effective treatment allows time for additional morbidity to develop.^17–19^

Limited data exist on the effects of early biologic treatment in patients with UC; however, in patients with Crohn’s disease, evidence suggests that early use of biologic treatment is associated with greater likelihood of response and remission and may delay disease progression and reduce treatment burden.^2,20^ Therefore, biologic intervention during an early stage of disease could modify disease course, prevent complications and hospitalization, and reduce healthcare costs.^17^ However, several barriers may delay the initiation of biologic treatment until after conventional therapies have failed, including issues with insurance reimbursement and prescriber or patient hesitation. Such delays may contribute to disease progression, complications, and increased healthcare costs.^2,17^

The RALEE study aimed to investigate the impact of early versus delayed initiation of vedolizumab in patients with IBD on treatment response and healthcare costs. Here, we report the impact on outcomes in patients with UC.

## Methods

### Objectives

The primary objectives of RALEE were to compare the proportion of patients with treatment response and the number of days from vedolizumab initiation to treatment response between early and delayed vedolizumab at 60 days (2 months), 6 months, and 12 months after initiating vedolizumab treatment. The exploratory objective was to compare healthcare costs between early and delayed vedolizumab 12 months after initiating vedolizumab treatment.

### Study Design

**Figure 1.**
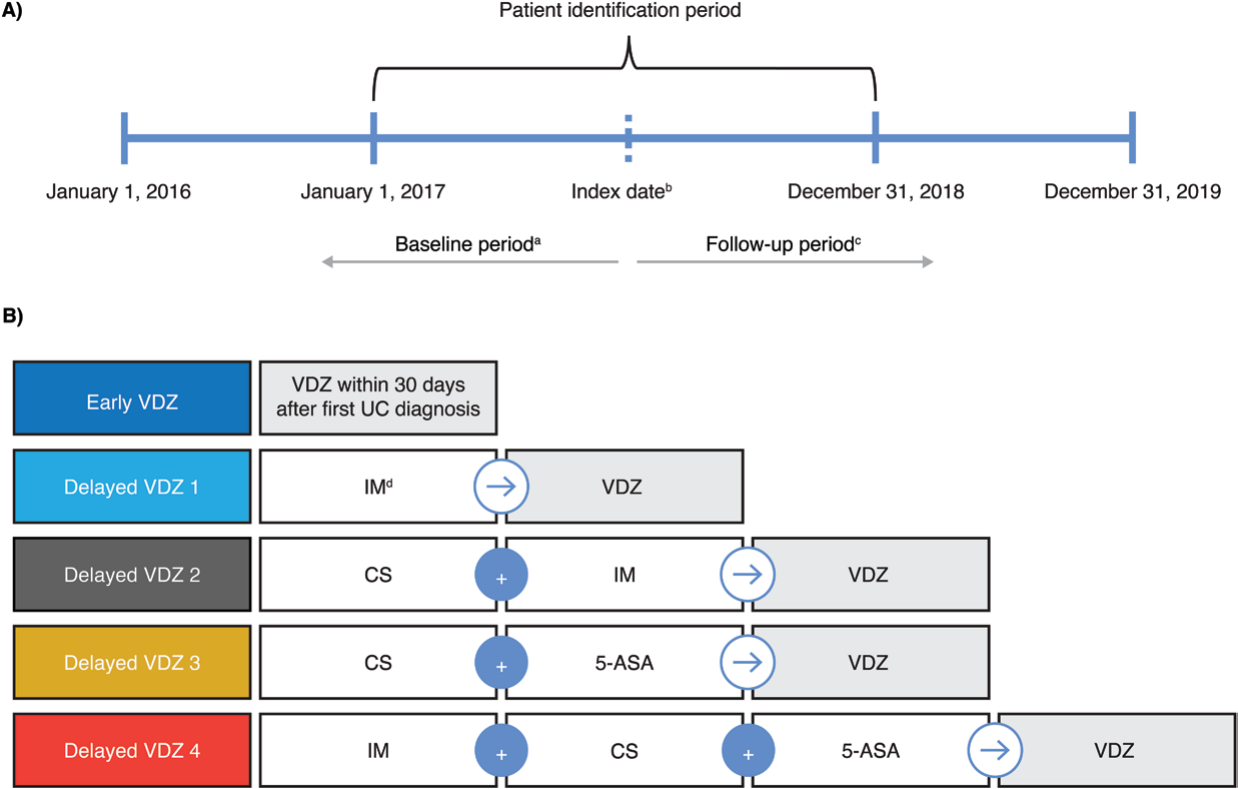
(A) Study design and (B) treatment groups. ^a^The baseline period was defined as the 12 months before the index date; ^b^the index date was defined as the first VDZ claim between January 1, 2017, and December 31, 2018; ^c^the follow-up period was defined as the period from the index date to the end of continuous enrollment, end of data cut (December 31, 2019) or death, whichever occurred first; ^d^Not 5-ASA. Abbreviations: 5-ASA, 5-aminosalicylate; CS, corticosteroid; IM, immunomodulator; UC, ulcerative colitis; VDZ, vedolizumab.

RALEE was a retrospective, observational, real-world study conducted using claims data from patients with UC treated with vedolizumab between January 1, 2016, and December 31, 2019 (Figure 1A). The index date was defined as the date of the first vedolizumab claim during the patient identification period (January 1, 2017, to December 31, 2018); the baseline period was defined as 12 months before the index date.

Patients were categorized into 5 post-diagnosis treatment groups: patients who received vedolizumab within 30 days of first UC diagnosis were included in the Early Vedolizumab group, whereas patients included in 1 of 4 Delayed Vedolizumab groups received vedolizumab more than 30 days after UC diagnosis and after treatment with corticosteroids, immunomodulators, and/or 5-ASA (Figure 1B).

Patients were followed from the index date until whichever occurred first: death, end of continuous enrollment, or December 31, 2019.

### Data Source

This study used the IBM® MarketScan® Commercial Claims and Encounters Database and the IBM MarketScan Medicare Supplemental and Coordination of Benefits Database, which contain de-identified patient healthcare data from inpatient, outpatient, and prescription claims in the United States.

### Study Population

Patients were eligible for the study if they met the following inclusion criteria (Supplementary Figure S1): aged 18 years or older on the index date; at least 2 separate diagnoses of UC within 6 months of the index date (International Classification of Diseases, ninth revision, clinical modification code 556.x; International Classification of Diseases, tenth revision, clinical modification code K51.x); continuous enrollment in the same healthcare plan for at least 12 months before and after the index date; at least one vedolizumab claim within the patient identification period; and a prescribed treatment sequence consistent with one of the prespecified treatment groups (Figure 1B).

Patients were excluded from the study if they had a diagnosis of both UC and Crohn’s disease within 6 months of the index date or if they had been exposed to vedolizumab, an antitumor necrosis factor-α treatment, or another biologic treatment in the 12 months before the index date.

### Baseline Variables

Baseline variables consisted of age group, age at first UC diagnosis, Charlson Comorbidity Index (CCI) score, insurance type, sex, US region, and year of diagnosis.

### Outcomes

The primary outcomes were the proportion of patients with a treatment response and the number of days from vedolizumab initiation to treatment response among the Early and Delayed Vedolizumab treatment groups, which was measured at 2, 6, and 12 months after vedolizumab initiation. Response was defined as no occurrence of any of the following events: new concomitant use of corticosteroids; IBD-related surgery; increased administration of vedolizumab (>900 mg of vedolizumab administered during the 8 weeks after vedolizumab initiation); vedolizumab treatment discontinuation (≥60 days without receiving vedolizumab); or a switch from vedolizumab to corticosteroids, immunomodulators, or 5-ASA.

Healthcare costs were stratified into drug, emergency department, home health, hospice, hospitalization, office-based, outpatient, skilled nursing facility, other, total healthcare, and total medical cost categories. These costs were measured from vedolizumab initiation in 6-month increments and reported as the per patient per month (PPPM) sum of patient and plan paid costs for each category. Costs were inflated to 2020 US dollars using the medical care component of the US Bureau of Labor Statistics Consumer Price Index.

### Statistical Analyses

Baseline demographics and clinical characteristics were analyzed for each treatment group as well as the overall cohort by calculating the proportion of patients in each category (categorical variables) or the median and interquartile range (continuous variables). A one-way analysis of variance was used to test for differences in age between treatment cohorts and chi-squared tests compared differences in other baseline characteristics.

For the remaining analyses, Delayed Vedolizumab 1-4 groups were combined into a single Delayed Vedolizumab group. The number of days from UC diagnosis to vedolizumab initiation was compared between responders and nonresponders at 2 months using the Wilcoxon rank-sum test. Association between treatment group (Early vs Delayed Vedolizumab) and response was evaluated at 2, 6, and 12 months after vedolizumab initiation using multivariate logistic regression (bivariate), with Early Vedolizumab as the reference group (adjusted for age, sex, US region, type of insurance, year of diagnosis, and CCI score). Association between the number of days from UC diagnosis to vedolizumab initiation and response was evaluated in the same model.

Healthcare costs were analyzed by calculating the mean (standard deviation) and median (interquartile range) cost PPPM for each type of claim and for total costs. Follow-up time for these analyses was 52 weeks; patients lost to follow-up were censored.

## Results

### Patient Attrition

In total, 1342 of 136 315 patients with UC who were identified from the databases met all eligibility criteria and were included in the analyses (Supplementary Figure S1).

### Baseline Demographics and Clinical Characteristics

Baseline demographics and clinical characteristics of the overall cohort and stratified by treatment group are shown in Table 1. The median age of patients in the overall cohort was 43.0 years, with a slightly higher proportion of male (51.0%) than female patients. Most patients were from the South of the United States (40.0%) and were commercially insured (96.4%). The majority of patients were diagnosed with UC in 2017 (86.4%; Table 1). The proportion of patients in each age group and the proportion of female patients were similar across treatment groups; however, more patients were aged 18-34 (37.1%) and female (53.9%) in the Early Vedolizumab group than in any of the Delayed Vedolizumab groups (Table 1).

**Table 1.** Patient demographic and baseline clinical characteristics.

Characteristic	Overall cohort (*n* = 1342)	Treatment group				
		Early VDZ (*n* = 89)	Delayed VDZ 1 (*n* = 101)	Delayed VDZ 2 (*n* = 199)	Delayed VDZ 3 (*n* = 505)	Delayed VDZ 4 (*n* = 448)
Age at first diagnosis (years) median (IQR)	43.0 (32.0-55.0)	38.0 (30.0-50.0)	42.0 (30.0-55.0)	42.0 (31.0-55.0)	43.0 (32.0-56.0)	45.0 (32.0-55.0)
Age group (years) *n* (%)						
18-34	412 (30.7)	33 (37.1)	35 (34.7)	61 (30.7)	157 (31.1)	126 (28.1)
35-44	290 (21.6)	22 (24.7)	19 (18.8)	48 (24.1)	109 (21.6)	92 (20.5)
45-54	293 (21.8)	19 (21.3)	21 (20.8)	40 (20.1)	98 (19.4)	115 (25.7)
55-64	293 (21.8)	10 (11.2)	24 (23.8)	47 (23.6)	113 (22.4)	99 (22.1)
≥65	54 (4.0)	5 (5.6)	2 (2.0)	3 (1.5)	28 (5.5)	16 (3.6)
CCI score						
Mean (SD)	0.36 (0.88)	0.27 (0.62)	0.46 (1.07)	0.40 (0.99)	0.32 (0.82)	0.37 (0.90)
Insurance type, *n* (%)						
Commercial	1294 (96.4)	86 (96.6)	99 (98.0)	197 (99.0)	479 (94.9)	433 (96.7)
Medicare	48 (3.6)	3 (3.4)	2 (2.0)	2 (1.0)	26 (5.1)	15 (3.3)
Sex, *n* (%)						
Male	685 (51.0)	41 (46.1)	56 (55.4)	101 (50.8)	263 (52.1)	224 (50.0)
Female	657 (49.0)	48 (53.9)	45 (44.6)	98 (49.2)	242 (47.9)	224 (50.0)
US region, *n* (%)						
North Central	329 (24.5)	20 (22.5)	29 (28.7)	43 (21.6)	135 (26.7)	102 (22.8)
Northeast	296 (22.1)	23 (25.8)	30 (29.7)	44 (22.1)	124 (24.6)	75 (16.7)
South	537 (40.0)	33 (37.1)	27 (26.7)	83 (41.7)	193 (38.2)	201 (44.9)
West	176 (13.1)	13 (14.6)	15 (14.9)	29 (14.6)	52 (10.3)	67 (15.0)
Unknown	4 (0.3)	0 (0.0)	0 (0.0)	0 (0.0)	1 (0.2)	3 (0.7)
Year of diagnosis, *n* (%)						
2017	1160 (86.4)	78 (87.6)	84 (83.2)	176 (88.4)	415 (82.2)	407 (90.8)
2018	182 (13.6)	11 (12.4)	17 (16.8)	23 (11.6)	90 (17.8)	41 (9.2)

Abbreviations: CCI, Charlson Comorbidity Index; IQR, interquartile range; SD, standard deviation; VDZ, vedolizumab.

Most patients received vedolizumab after treatment with one or more conventional therapies, including corticosteroids, immunomodulators, and 5-ASA; only 6.6% of patients initiated vedolizumab treatment early after diagnosis (Supplementary Figure S2A).

### Treatment Response

**Figure 2.**
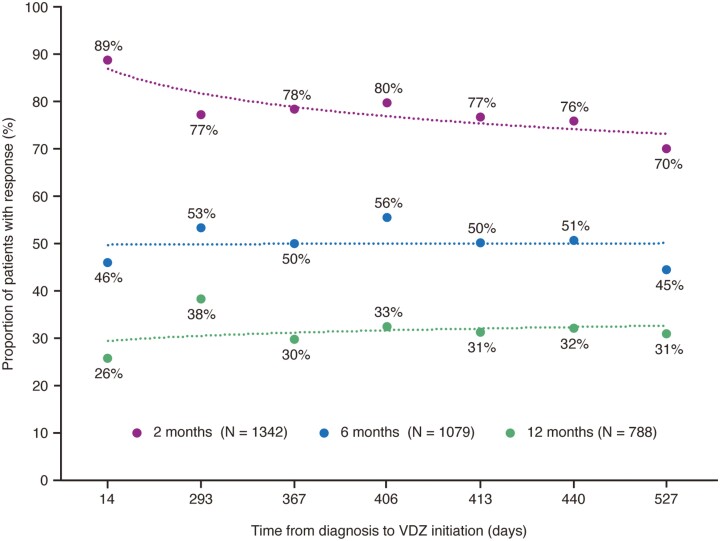
Proportion of patients who responded to VDZ at 2, 6, and 12 months by time from diagnosis to VDZ treatment initiation. Abbreviation: VDZ, vedolizumab.

The proportion of patients who responded to treatment within 2 months of vedolizumab initiation was higher for Early Vedolizumab (88.8%) than any of the Delayed Vedolizumab groups (70.1%-79.8%; Supplementary Figure S2A). The proportion of patients who responded to treatment within 2 months decreased as the time from UC diagnosis to vedolizumab initiation increased; however, the proportion of patients who responded to treatment within 6 and 12 months remained similar as the time from UC diagnosis and treatment response increased (Figure 2). Patients with a treatment response within 2 months of initiating vedolizumab received vedolizumab on average 226 days earlier after UC diagnosis than those who had nonresponse (*P *< .001; Supplementary Figure S3).

**Figure 3.**
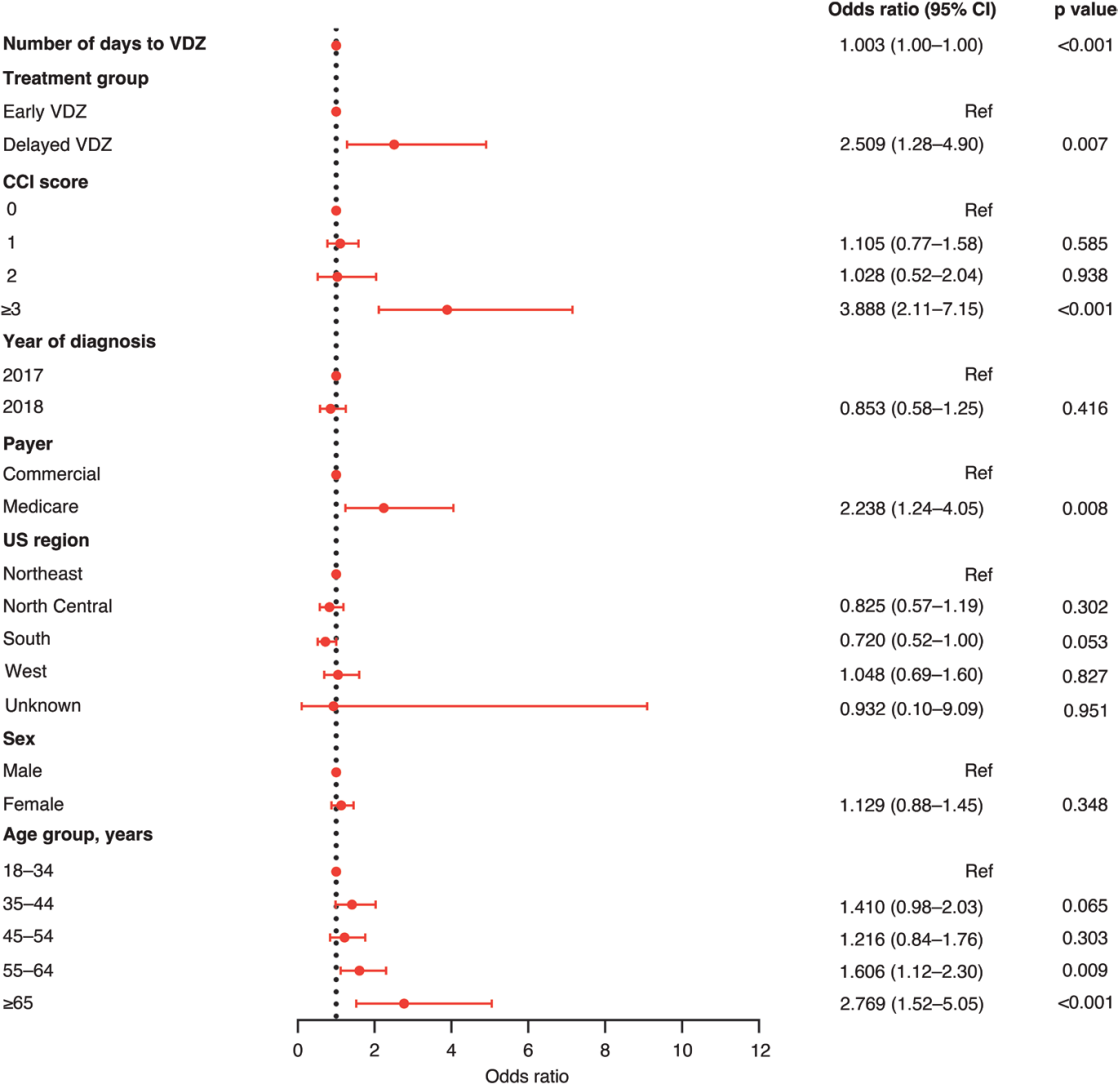
Adjusted odds ratios for nonresponse to VDZ 2 months after VDZ initiation. Abbreviations: CCI, Charlson Comorbidity Index; CI, confidence interval; Ref, reference; VDZ, vedolizumab.

**Figure 4.**
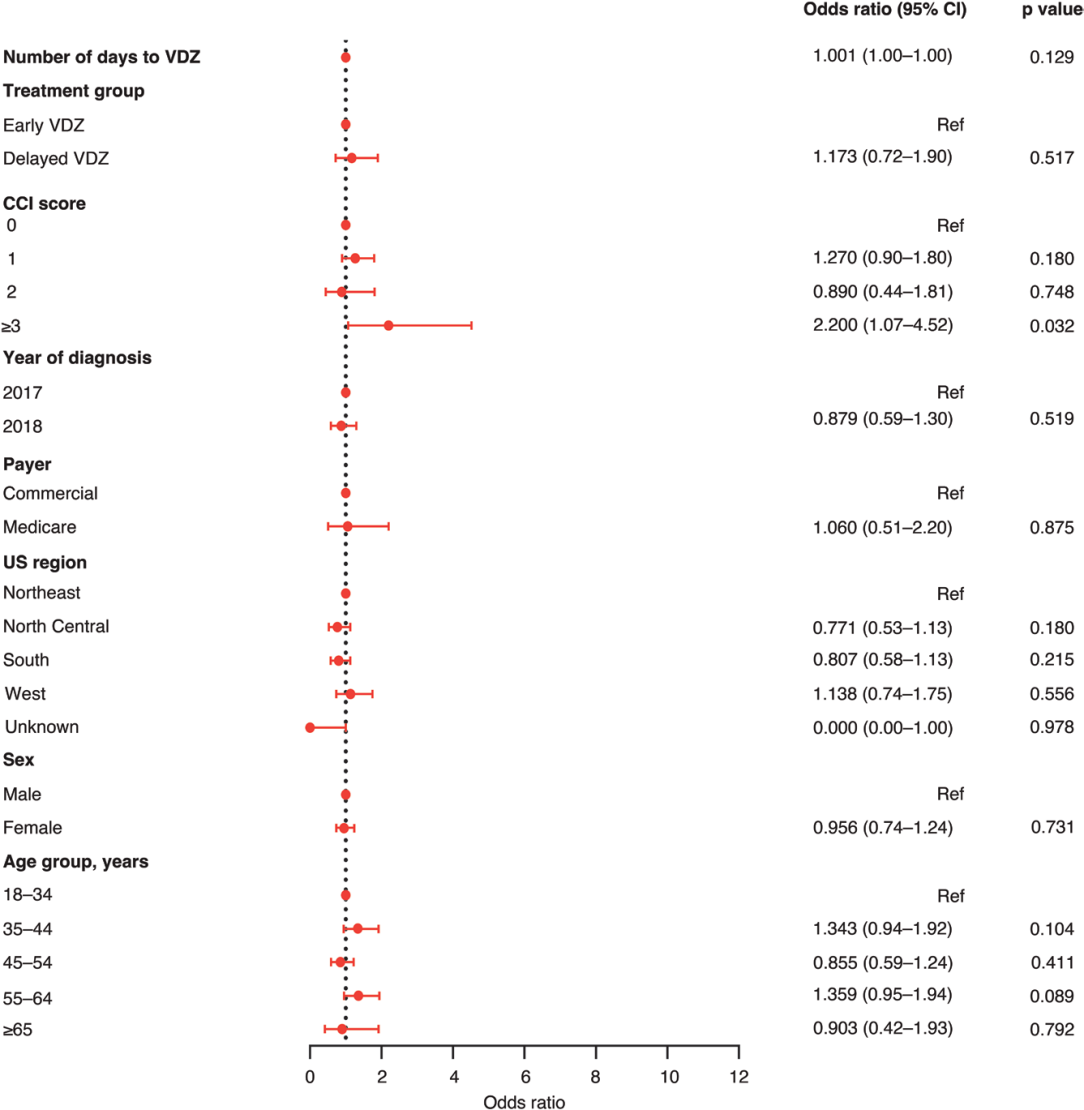
Adjusted odds ratios for nonresponse to VDZ 6 months after VDZ initiation. Abbreviations: CCI, Charlson Comorbidity Index; CI, confidence interval; Ref, reference; VDZ, vedolizumab.

**Figure 5.**
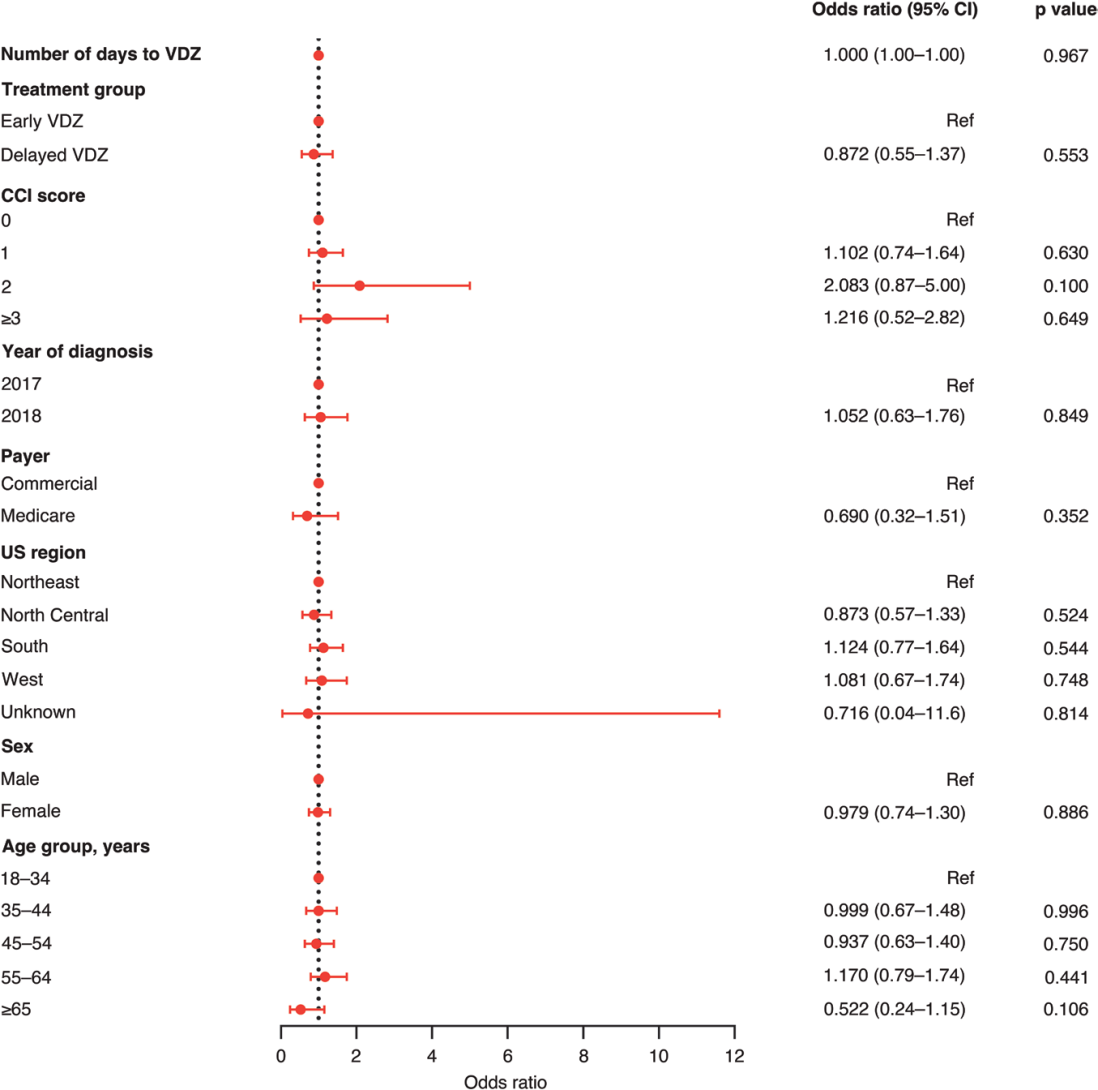
Adjusted odds ratios for nonresponse to VDZ 12 months after VDZ initiation. Abbreviations: CCI, Charlson Comorbidity Index; CI, confidence interval; ref, reference; VDZ, vedolizumab.

In adjusted analyses, Delayed Vedolizumab (odds ratio [OR], 2.51; 95% confidence interval [CI], 1.28-4.90), age of 65 years or older (OR, 2.77; 95% CI, 1.52-5.05), a CCI score of 3 or more (OR, 3.89; 95% CI, 2.11-7.15), and a higher number of days from UC diagnosis to vedolizumab initiation (OR, 1.00; 95% CI, 1.00-1.00) were associated with significantly higher odds of nonresponse within 2 months after vedolizumab initiation (Figure 3). A CCI score of 3 or more (OR, 2.20; 95% CI, 1.07-4.52) was the only variable associated with significantly higher odds of nonresponse within 6 months after vedolizumab initiation (Figure 4). None of the variables included in the model were associated with treatment nonresponse within 12 months after vedolizumab initiation (Figure 5).

### Healthcare Costs

Healthcare costs PPPM in the 12 months after initiating vedolizumab for the overall cohort, Early Vedolizumab group, and Delayed Vedolizumab group are shown in Table 2. Mean drug costs were lower in the Early Vedolizumab group ($1183) than the Delayed Vedolizumab group ($1780); however, mean hospitalization costs, outpatient costs, and office-based costs were higher in the Early Vedolizumab group than in the Delayed Vedolizumab group. In the overall cohort, mean emergency department costs, hospice costs, and skilled nursing facility costs were negligible ($0, $1, and $4, respectively). Mean total healthcare costs were $5897 in the Delayed Vedolizumab group and $6492 in the Early Vedolizumab group.

**Table 2.** Healthcare costs for the overall cohort, and Early VDZ and Delayed VDZ treatment groups.

	Overall cohort (*n* = 1342)	Early VDZ (*n* = 89)	Delayed VDZ (*n* = 1253)
Drug costs PPPM, US$			
Mean (SD)	1740 (2095)	1183 (2150)	1780 (2086)
Median (IQR)	1111 (383-2422)	415 (136-959)	1201 (416-2464)
Emergency department costs PPPM, US$			
Mean (SD)	0 (0)	0 (0)	0 (0)
Median (IQR)	0 (0-0)	0 (0-0)	0 (0-0)
Home health costs PPPM, US$			
Mean (SD)	248 (729)	285 (899)	246 (716)
Median (IQR)	0 (0-16)	0 (0-16)	0 (0-16)
Hospice costs PPPM, US$			
Mean (SD)	1 (15)	0 (2)	1 (15)
Median (IQR)	0 (0-0)	0 (0-0)	0 (0-0)
Hospitalization costs PPPM, US$			
Mean (SD)	776 (1986)	960 (2108)	763 (1977)
Median (IQR)	0 (0-677)	0 (0-471)	0 (0-677)
Office-based costs PPPM, US$			
Mean (SD)	1404 (1546)	1527 (1799)	1395 (1526)
Median (IQR)	827 (187-2242)	382 (133-2840)	854 (191-2232)
Outpatient costs PPPM, US$			
Mean (SD)	1668 (2628)	2456 (3077)	1612 (2586)
Median (IQR)	559 (228-1919)	977 (296-4242)	550 (228-1824)
Skilled nursing facility costs PPPM, US$			
Mean (SD)	4 (45)	0 (0)	4 (46)
Median (IQR)	0 (0-0)	0 (0-0)	0 (0-0)
Other costs PPPM, US$			
Mean (SD)	12 (124)	7 (32)	12 (128)
Median (IQR)	0 (0-0)	0 (0-0)	0 (0-0)
Total healthcare costs PPPM, US$			
Mean (SD)	5936 (4161)	6492 (3599)	5897 (4197)
Median (IQR)	5087 (3474-7283)	5437 (4236-7893)	5050 (3453-7257)
Total medical costs PPPM, US$			
Mean (SD)	4196 (3667)	5309 (3406)	4117 (3673)
Median (IQR)	3300 (2032-5150)	4613 (3221-6373)	3216 (1979-5039)

Abbreviations: IQR, interquartile range; PPPM, per patient per month; SD, standard deviation; VDZ, vedolizumab.

## Discussion

This study compared the impact of early versus delayed initiation of vedolizumab on treatment response, with specific focus on treatment sequence and time since UC diagnosis. Patients who received vedolizumab within 30 days of UC diagnosis were significantly less likely to experience nonresponse within the 2 months after initiating vedolizumab treatment than those for whom vedolizumab was delayed until after treatment with corticosteroids, immunomodulators, and/or 5-ASA. Furthermore, a greater number of days between UC diagnosis and vedolizumab treatment initiation was significantly associated with a higher likelihood of nonresponse within 2 months after initiating vedolizumab treatment. These findings suggest that administering vedolizumab as early as possible after UC diagnosis may increase the likelihood of treatment response compared with delaying initiation of vedolizumab until after conventional therapies (5-ASAs, corticosteroids, and immunomodulators).

Response to vedolizumab may be influenced by a number of factors. For instance, clinical trial data have demonstrated an exposure–response relationship for vedolizumab.^21–23^ A phase 4 trial evaluating the effect of dose optimization in patients with early nonresponse to vedolizumab and high drug clearance concluded that although dose optimization may not be necessary for this subpopulation, duration of treatment, disease activity, and systemic inflammation may affect response.^24^ Here, we demonstrate that time to treatment initiation may also be included as a determinant of treatment response.

In contrast to the results of our study, 2 retrospective studies found no benefit of early intervention in patients with UC.^25,26^ The first investigated early treatment with adalimumab or infliximab on the risk of colectomy, hospitalization, or secondary loss of response in patients with UC and reported no improvement on risk in the early versus delayed treatment groups; however, patients who received early treatment generally had more severe disease than those who initiated treatment later. Therefore, disease severity could have affected these outcomes rather than early versus delayed treatment initiation.^25^ The second study compared the rates of clinical remission, corticosteroid-free remission, and endoscopic remission among patients with different stages of Crohn’s disease or UC receiving treatment with vedolizumab.^26^ Disease duration was not a predictor of treatment response to vedolizumab in patients with UC, but it was in patients with early Crohn’s disease (≤2 years) compared with those who had been diagnosed with Crohn’s disease more than 2 years before.^26^

We expand on the published literature and show that early treatment with vedolizumab after UC diagnosis is associated with an increased likelihood of achieving a treatment response within 2 months after treatment initiation compared with delayed initiation of vedolizumab, which is consistent with the American Gastroenterological Association clinical guidelines that recommend early use of biologics, rather than after failure of 5-ASA, for adult patients with moderately to severely active UC.^27^ However, within 6 and 12 months after initiating vedolizumab, we found that patients who received vedolizumab early after UC diagnosis were as likely to experience nonresponse than those who had delayed initiation. Although this may suggest that early initiation of vedolizumab does not continue to exert beneficial effects on treatment response after a prolonged period of treatment, it is likely that at least some of the events that were used to measure response in this study, such as vedolizumab discontinuation, accumulated over time and reduced the response to vedolizumab recorded at 6 and 12 months.

In general, mean total healthcare costs were similar in the Early and Delayed Vedolizumab groups, although there were small differences in costs from different types of claims, such as lower drug costs in the Early Vedolizumab group. The impact of biologics on healthcare costs is not fully understood; however, our findings contradict the perception that early initiation of vedolizumab treatment may be more expensive than if it was delayed until after conventional therapy.^28–30^ Indeed, a commission on the cost of IBD concluded that direct increases in healthcare costs should be assessed considering the potential to improve disease management and therefore reduce indirect costs associated with poorly controlled disease.^31^ Healthcare costs were assessed 12 months after initiating vedolizumab, at which point there was no difference in the odds of nonresponse between the Early Vedolizumab and Delayed Vedolizumab groups; therefore, it is possible that the effects of receiving early vedolizumab on treatment response and healthcare costs are evident before 12 months of treatment.

It is important to consider the limitations of this analysis when interpreting the results. The definition of treatment response used in this analysis was based on the absence of specific clinical events recorded in the databases and was not assessed via measures typically used in clinical trials of UC, such as endoscopy or symptoms.^32^ As a result, it is possible that some cases of clinical treatment response were missed or included erroneously as false positive results. However, our definition of treatment response included new concomitant use of corticosteroids and incidence of IBD-related surgery; the goal of treatment for UC is corticosteroid-free remission and prevention of surgery, and therefore these events can be considered a proxy for response to treatment.^16^ Similarly, we included dose escalation, discontinuation, and treatment switching in the response definition. These are all strategies recommended when a patient has no response or loses response to their biologic therapy.^33,34^ Therefore, although the lack of endoscopy and symptom data in claims databases limits how treatment response can be defined, the definition used here encompasses multiple factors associated with treatment response in real-world settings.

The use of claims data also limited the scope of our analyses. For example, although the Early Vedolizumab group received vedolizumab within 30 days of UC diagnosis, these patients may not have been at an early stage of disease at the point of diagnosis. This should be taken into consideration when drawing conclusions, although this limitation is shared by other observational database studies, which are restricted by the data available. Similarly, owing to the difficulty of establishing disease severity from claims data, we were unable to adjust for this and other clinical characteristics. Patients in the Early Vedolizumab group were also, on average, slightly younger at baseline than patients in the Delayed Vedolizumab groups and had a lower mean CCI score, which could suggest a different inflammatory process related to earlier onset of disease. Furthermore, the Early Vedolizumab group accounted for a relatively small proportion of the study cohort. This group may also be a possible source of selection bias because patients receiving early treatment with vedolizumab may be more likely to be those who received vedolizumab in a tertiary referral or academic center. Lastly, these results are generalizable to commercially insured adult US patients with UC but may not be representative of the general US patient population.

## Conclusions

The results of this analysis suggest that patients with UC are more likely to respond to treatment within 2 months of initiating vedolizumab if treatment is initiated early after UC diagnosis than if vedolizumab treatment is delayed until after conventional therapy. Furthermore, early initiation with vedolizumab does not increase healthcare costs after 12 months compared with delayed initiation of vedolizumab after conventional therapy. Therefore, the early initiation of vedolizumab could improve outcomes in patients with UC by increasing the number of patients with a treatment response to a disease-modifying therapy. Clinicians should not delay effective therapy and should embrace early use of vedolizumab in patients with moderately to severely active UC.

## Supplementary Material

otae061_suppl_Supplementary_Figures_S1-S3

## Data Availability

Data not publicly available.
