# Supplementary material for: Real-World Treatment Outcomes Associated With Early Versus Delayed Vedolizumab Initiation in Patients With Ulcerative Colitis
Source: Crohns Colitis 360. 2024 Oct 22;6(4):otae061. doi: 10.1093/crocol/otae061 (PMC11535256; doi:10.1093/crocol/otae061)
Supplement: otae061_suppl_Supplementary_Figures_S1-S3 [file otae061_suppl_supplementary_figures_s1-s3.docx]

# Supplementary Material

## Supplementary Figure 1. Patient attrition.


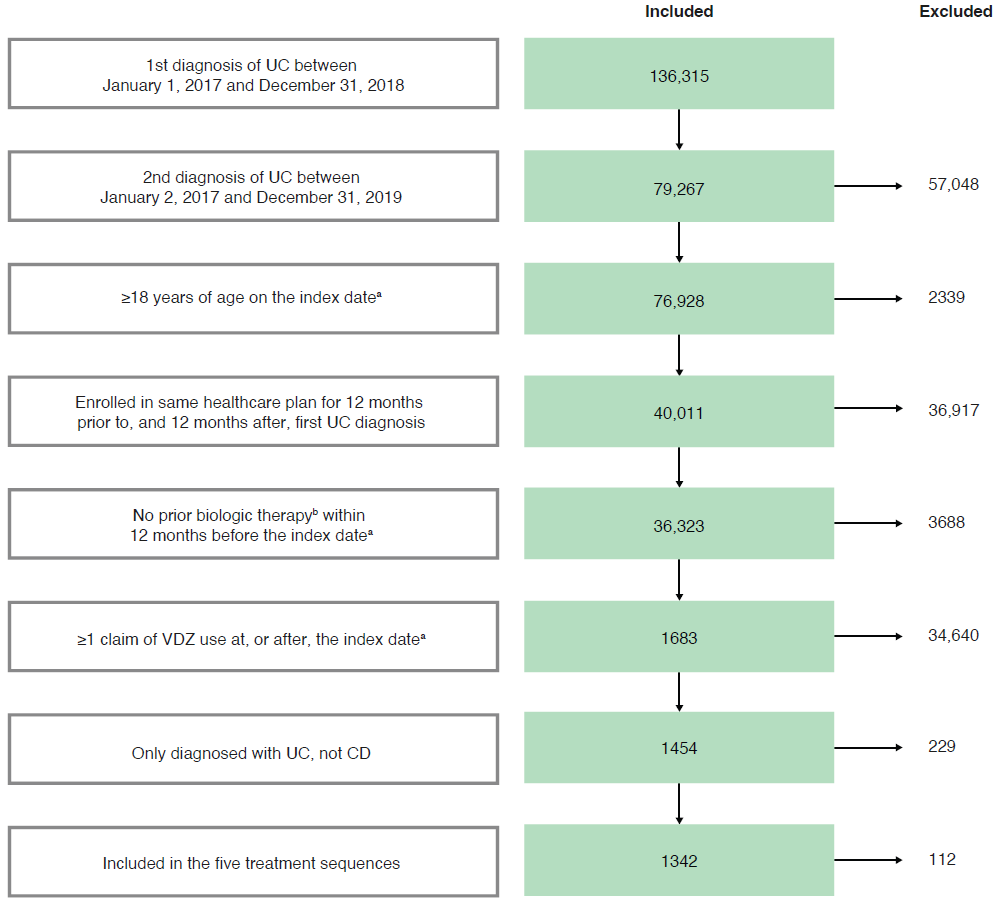


^a^Date of first UC diagnosis; ^b^Biologics included adalimumab, certolizumab, infliximab, natalizumab, ustekinumab, and vedolizumab. CD, Crohn’s disease; UC, ulcerative colitis; VDZ, vedolizumab.

## Supplementary Figure 2. A) Proportion of patients in each treatment group and B) proportion of patients who responded to VDZ in the overall cohort and by treatment group 2 months after VDZ treatment initiation.


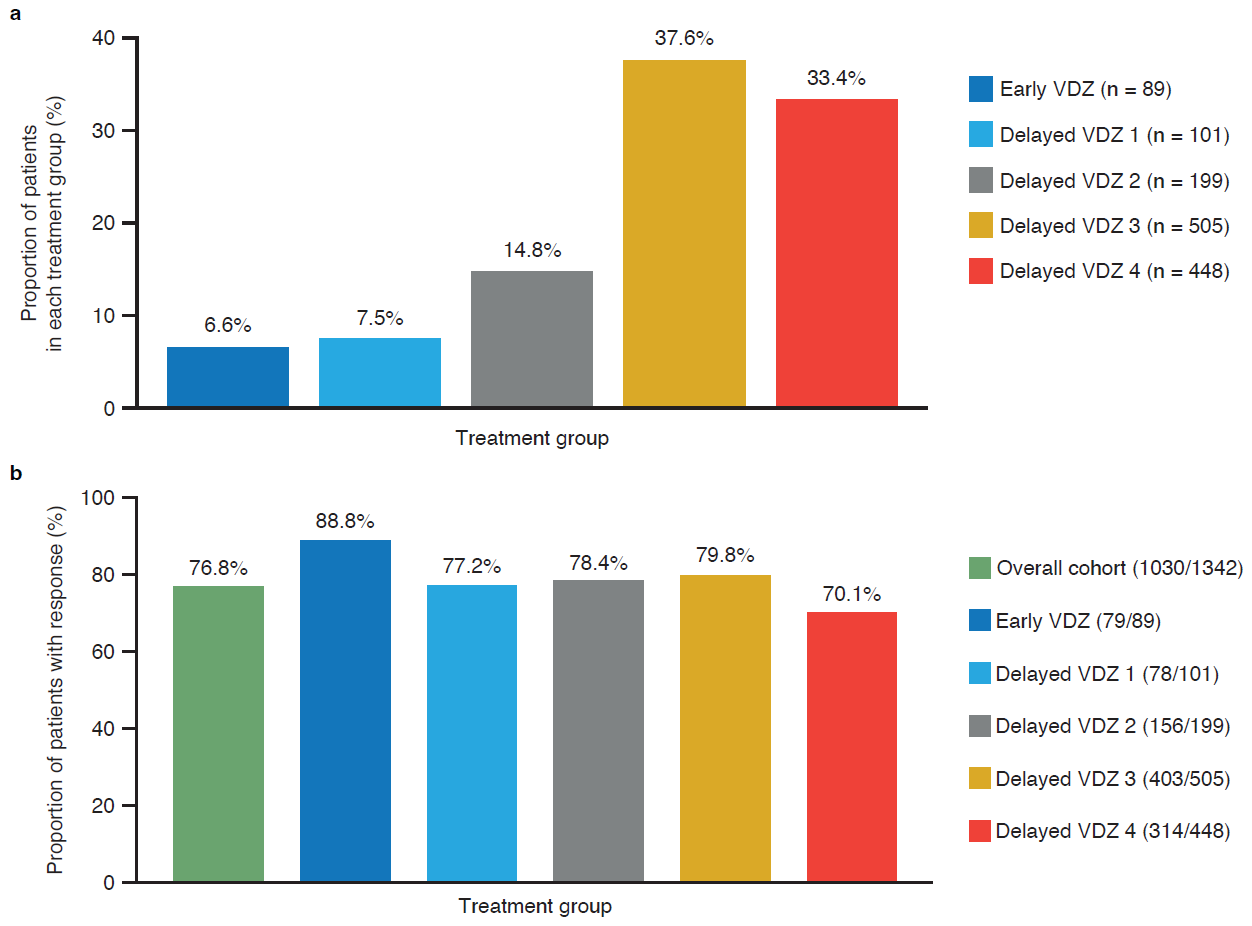


Values may not sum to 100% due to rounding. Early VDZ group received VDZ within 30 days of diagnosis; Delayed VDZ 1 group received IM before VDZ; Delayed VDZ 2 received CS and IM before VDZ; Delayed VDZ 3 received CS and 5-ASA before VDZ; Delayed VDZ 4 received IM, CS and 5-ASA before VDZ. 5-ASA, 5-aminosalicylate; CS, corticosteroid; IM, immunomodulator; VDZ, vedolizumab.

## Supplementary Figure 3. Number of days from UC diagnosis to VDZ initiation for responders A) and nonresponders B) at 2 months.


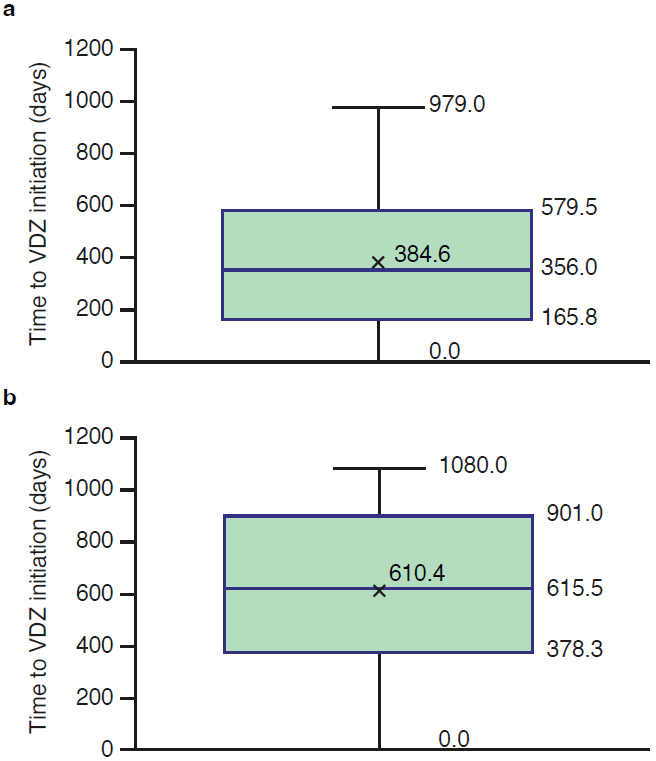


VDZ, vedolizumab.
